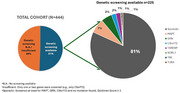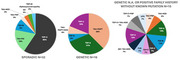# Genetic and Pathological Characteristics of Frontotemporal Dementia with Right Anterior Temporal Predominance: A Multicenter Retrospective Cohort Study

**DOI:** 10.1002/alz70857_103987

**Published:** 2025-12-25

**Authors:** Hulya Ulugut, Kyan Younes, Maxime Bertoux, Maxime Montembeault, Giorgio G Fumagalli, Alma Ghirelli, Edoardo G. Spinelli, Na‐Yeon Jung, Bedia Samancı, Ignacio Illán‐Gala, Jennifer Thompson, Christopher Kobylecki, Alexander Santillo, Elisabet Englund, María Landqvist Waldö, Lina Riedl, Jan Van den Stock, Mathieu Vandenbulcke, Rik Vandenberghe, Robert Laforce, Simon Ducharme, Peter S. Pressman, Carmela Tartaglia, Paulo Caramelli, Leonardo Cruz de Souza, Leonel Tadao Takada, Alexandre Morin, Matthias L Schroeter, Eun‐Joo Kim, So Young Moon, Federica Agosta, Elisa Canu, Massimo Filippi, Hakan Gurvit, Janine Diehl‐Schmid, Daniela Galimberti, Thibaud Lebouvier, Bruce L. Miller, Virginia E. Sturm, Toji Miyagawa, Jennifer L. Whitwell, Brad F. Boeve, Jonathan D. Rohrer, Maria Luisa Gorno Tempini, Keith A. Josephs, Julie S Snowden, Jason D Warren, Katherine P Rankin, Yolande A.L. Pijnenburg, International rtvFTD Working Group

**Affiliations:** ^1^ Memory & Aging Center, Department of Neurology, University of California in San Francisco, San Francisco, CA, USA; ^2^ Department of Neurology and Neurological Sciences, Stanford University, Stanford, CA, USA; ^3^ Lille Neuroscience & Cognition, Inserm, Univ. Lille, CHU Lille, LiCEND & DistALZ, Lille, France; ^4^ Douglas Mental Health University Institute, Montréal, QC, Canada; ^5^ University of Trento, Rovereto, Italy, Rovereto, Italy; ^6^ Vita‐Salute San Raffaele University, Milan, Italy; ^7^ IRCCS San Raffaele Scientific Institute, Milan, MI, Italy; ^8^ Pusan National University Yangsan Hospital, Yangsan, Korea, Republic of (South); ^9^ Istanbul Faculty of Medicine, Istanbul University, Istanbul, Turkey; ^10^ Sant Pau Memory Unit, Hospital de la Santa Creu i Sant Pau ‐ Biomedical Research Institute Sant Pau ‐ Universitat Autònoma de Barcelona, Barcelona, Barcelona, Spain; ^11^ University of Manchester, Manchester, Lancashire, United Kingdom; ^12^ The University of Manchester, Manchester, n.a., United Kingdom; ^13^ Clinical Memory Research Unit, Lund University, Malmö, Sweden; ^14^ Skåne University Hospital, Lund, Sweden; ^15^ Lund University, Lund, Sweden; ^16^ Technical University of Munich, School of Medicine, Munich, Germany; ^17^ KU Leuven, Leuven, Belgium; ^18^ Alzheimer Research Centre, Leuven Brain Institute, KU Leuven, Leuven, Belgium; ^19^ Laboratory for Cognitive Neurology, Department of Neurosciences, KU Leuven, Leuven, ‐, Belgium; ^20^ Research Chair on Primary Progressive Aphasia ‐ Fondation de la famille Lemaire, Quebec, QC, Canada; ^21^ Montreal Neurological Institute, McGill University, Montreal, QC, Canada; ^22^ Layton Healthy Aging and Alzheimer's Disease Research Center, Portland, OR, USA; ^23^ University of Toronto Department of Medicine, Toronto, ON, Canada; ^24^ Universidade Federal de Minas Gerais, Belo Horizonte, MG, Brazil; ^25^ Federal University of Minas Gerais, Belo Horizonte, Minas Gerais, Brazil; ^26^ Hospital das Clínicas, University of Sao Paulo Medical School, São Paulo, São Paulo, Brazil; ^27^ Department of Neurology, Rouen University Hospital, F‐76000, Rouen, FR, Paris, France; ^28^ Max Planck Institute for Human Cognitive and Brain Sciences, Leipzig, Germany; ^29^ Pusan Medical University Hospiral, Pusan, Korea, Republic of (South); ^30^ Ajou University School of Medicine, Suwon, Gyeonggi‐do, Korea, Republic of (South); ^31^ Vita‐Salute San Raffaele University, Milan, MI, Italy; ^32^ IRCCS San Raffaele Scientific Institute, Milan, Italy; ^33^ Behavioural Neurology and Movement Disorders Unit, Department of Neurology, Istanbul Faculty of Medicine, Istanbul University, Istanbul, Turkey, Istanbul, Turkey; ^34^ Inn‐Salzach‐Klinikum, Wasserburg, Germany; ^35^ University of Milan, Milan, MI, Italy; ^36^ Univ. Lille, Inserm, CHU Lille, Lille Neuroscience & Cognition, UMR‐S 1172, Lille, Hauts‐de‐France, France; ^37^ Memory and Aging Center, Weill Institute for Neurosciences, University of California, San Francisco, San Francisco, CA, USA; ^38^ University of California San Francisco, San Francisco, CA, USA; ^39^ Mayo Clinic, Rochester, MN, USA; ^40^ Department of Neurology, Mayo Clinic, Rochester, MN, USA; ^41^ Dementia Research Centre, Queen Square Institute of Neurology, University College London, London, ‐, United Kingdom; ^42^ Department of Neurology, Memory and Aging Center, University of California San Francisco, San Francisco, CA, USA; ^43^ Institute of Brain, Behaviour and Mental Health, University of Manchester, Manchester, United Kingdom; ^44^ Dementia Research Centre, UCL Queen Square Institute of Neurology, London, London, United Kingdom; ^45^ Memory and Aging Center, Weill Institute for Neurosciences, University of California San Francisco, San Francisco, CA, USA; ^46^ Alzheimer Center Amsterdam, Department of Neurology, Amsterdam UMC, location VUmc, Amsterdam, Netherlands

## Abstract

**Background:**

Frontotemporal dementia (FTD) with right anterior temporal lobe (RATL) predominant atrophy is an emerging area of interest. Recent findings by the International Working Group (IWG) have identified this subtype as having a distinct clinical profile within the FTD spectrum (Ulugut et al., 2024, A&D). However, its genetic and pathological underpinnings remain unexplored in large, multicultural cohorts.

**Methods:**

Retrospective analyses encompassing clinical, genetic, pathological, and neuroimaging data from 23 IWG sites across 13 countries in Asia, Middle East, Europe, North and South America were conducted. The study included 444 patients with FTD exhibiting predominant RATL atrophy.

**Results:**

Genetic screening was performed on 51% (*n* = 225) of the cohort for at least the major frontotemporal lobar degeneration (FTLD) mutations including microtubule‐associated protein tau (*MAPT*), progranulin (*GRN*), and chromosome 9 open reading frame 72 (*C9orf72*), or dementia panels encompassing extended sets of genes. Of these, 81% were sporadic, showing negative results for the screened genes and a modified Goldman Score of ≥ 3, indicating a negative family history for dementia. The *MAPT* mutation was the most common genetic variant, identified in 7% of the screened cases (Figure 1). Pathological confirmation was available for 63 patients. Among the sporadic cases, transactive response DNA‐binding protein 43 type C (TDP‐C) pathology was most prevalent (60%, *n* = 32), while tau‐MAPT pathology was most common in the genetic cases (38%, *n* = 16). Fifteen cases did not fit neatly into genetic or sporadic categories, displaying heterogeneous pathologies (Figure 2). At the initial visit, compared to genetic cases, patients with TDP‐C pathology were older, and more frequently exhibited semantic deficits, with less frequent attention difficulties and executive dysfunction. No differences were observed in sex distribution, symptom duration or disease severity between genetic and sporadic TDP‐C cases. However, left handedness was more common in TDP‐C cases (14%) compared to genetic cases (5%).

**Conclusion:**

While FTD with RATL atrophy primarily appears sporadic, a significant proportion of cases exhibit genetic variants. These sporadic and genetic subtypes display distinct neuropathological features and clinical manifestations. Given the implications for therapeutic strategies, precise clinical and molecular subtyping is critical for enhancing patient management and ensuring appropriate enrollment in clinical trials.